# The amount of antioxidants in honey has a strong relationship with the plants selected by honey bees

**DOI:** 10.1038/s41598-023-51099-9

**Published:** 2024-01-03

**Authors:** Zahra Shakoori, Elham Salaseh, Ahmad Reza Mehrabian, Dariush Minai Tehrani, Niluofar Famil Dardashti, Farid Salmanpour

**Affiliations:** 1 Department of Plant Sciences and Biotechnology, Faculty of Life Sciences and Biotechnology, Shahid Beheshti University, Tehran, Iran; 2 Department of Biodiversity and Ecosystem Management, Environmental Sciences Research Institute, Shahid Beheshti University, Tehran, Iran; 3 Department of Chemistry, Faculty of Pharmaceutical Chemistry, Tehran Medical Sciences, Islamic Azad University, Tehran, Iran

**Keywords:** Biochemistry, Ecology, Plant sciences

## Abstract

As one of the main sources of natural antioxidants, flowering plants play a role in the prevention and treatment of many diseases directly and indirectly. Honey is considered as an important nutrient in the supply of natural antioxidants, the amount of which is directly dependent on the plant origin and geographical location of the bee feeding place. The existence of valuable communities of native and endemic plant species has turned Alborz, Zagros and Azerbaijan into the most important hubs of honey production in Iran. In this study, we collected samples of honey from more than 90 regions in Alborz, Zagros and Azerbaijan during the years 2020 to 2021. We evaluated the samples using melissopalynology method and measuring the amount of antioxidant activity. The rise of antioxidant activity in honey is dependent on the abundance of some plant families as well botanical origins. The abundance of plant families Rosaceae, Amaranthaceae, Fabaceae and Asteraceae showed a higher influence on the amount of antioxidants in honey than other plant families. Also, the abundance of plant families Rosaceae and Fabaceae increased with increasing altitude. In general, the amount of antioxidant activity of honey samples shows a different percentage under the influence of ecological and geographical changes.

## Introduction

Today, all kinds of diseases, especially cancers, are a serious concern for modern humans in terms of their abundance, existence of different types and the response to treatment^1^. Cancer is a disease that can be caused by the imbalance between antioxidants and reactive oxygen species^2^. Therefore, a preventive action for diseases in the first place can greatly reduce the possibility of contracting this disease and also provide the possibility of treating it with less side effects and longer survival^3^. The key role of natural antioxidants in biological systems and their relationship with the treatment and prevention of various diseases has been the focus of nutrition and health researchers^4,5^. Natural antioxidants in food have shown positive effects in the prevention and treatment of many types of cancer^6,7^. It also prevents metabolic disorders by eliminating free radicals^8,9^. Antioxidants in food are generally secondary metabolites of plants, whose activity level is influenced by the chemical structure of their constituents^10,11^. Therefore, green plants are considered as important food sources containing natural antioxidants in the human diet^12^.

Honey is considered as a valuable food that is rich in natural antioxidants^13^. The amount of antioxidants in honey depends on several factors such as harvest season, humidity, the type of bee and most importantly, the type of plants that the bee fed on^14–16^. The vegetable origin of honey is considered the most determining parameter for the amount of antioxidants in honey^17,18^. Therefore, the place of honey production can determine the amount of antioxidants due to the type of plants in that area^19,20^. Plants have been used as food and medicine since the past, and today they are the basis of many commercial medicines^21,22^. Molecular studies have shown that plants have various medicinal properties due to their different chemical compounds^23^. Meanwhile, some plants specifically contain more antioxidants^24^. Plants have pharmacological effects on humans due to the production of some chemical substances, among these compounds are phenolic compounds, alkaloids and flavonoids, which are also known as secondary metabolites^25,26^. Medicinal plants can be used in the prevention or treatment of some diseases due to their antioxidant and phenolic compounds^27^.

Asteraceae is a large family of flowering plants with more than 2500 species and 1600 genera. The plants of this family have the ability to grow in most habitats due to their high adaptability and are scattered in different parts of the world except Antarctica. Among the important plants of this family, we can mention chicory, daisies and dandelion. Many plants of the Asteraceae family contain amounts of polyphenol compounds, antioxidants, and flavonoids that increase biological activity and protect the consuming organisms against oxidative stress conditions. Therefore, the plants of this family are considered as a useful source of vitamins, antioxidants, polyphenol compounds and proteins^27,28^. Amaranthaceae family, known as Amaranthus, which is found mainly in the form of leafy vegetables in the world, is considered as a good source of antioxidants^29^. The plants of this large family are not only flexible against adverse environmental conditions such as lack of water, salinity, dryness and poor soil, but also increase the amount of flavonoids, phenolic acids, vitamins and natural antioxidants^30,31^. This family contains more than 2000 plant species and 175 genera that are found in cold, temperate and tropical regions. A series of plants of the Amaranthaceae family can be used as herbal medicine in the world due to the presence of phytochemical properties such as phenolic compounds, terpenoids and betalains^32^. Fabaceae as one of the largest plant families has about 19,500 species and 770 genera^33,34^. The plants of this family with chemical compounds such as flavonoids, alkaloids, lectins and phenolic acids can have antioxidant, anti-inflammatory, anti-ulcer, anti-rheumatic and anti-cancer properties and activities^35^. Therefore, in today's world, plants of this family can be used in paint, insecticide, fuel, etc. industries^36^. The Rosaceae family, known as roses, is one of the largest plant families, including about 120 genera and 4000 species^37^.

Approximately 7800 plant taxa comprise the flora of Iran^38^, about 2700 of which are confined to the geographical boundaries of Iran^39^. The climatological diversity, history of vegetation, geographical isolation^40^, complex tectonic history, special soils^41^, and intersection of diverse phytogeographical units^42^ lead to the high potential of this region for diversification and make it a global center of diversity for plants^43^ and one of the most important distribution centers of crop wild relatives in the world^44^. Accordingly, a wide range of nectar and pollen producing plants were distributed in Iran which has led to the production of about 55 different types of mono-floral and polyfloral honey in Iran^45^, a wide range of which are classified as medical food and medical grades^46^. Melissopalynology is considered as an effective method of identifying and determining the abundance of plants selected by honey bees, which, along with measuring the antioxidant activity of honey, can determine the effect of plants on the antioxidant activity of honey^47,48^. In this study, to determine the frequency of the presence of plant families of the antioxidant activity of honey, the method of melissopalynology and the laboratory measurement of the antioxidant activity of honeys produced in the Zagros and Alborz Mountains were used. Samples (unrefined and non-clay honeys) were collected directly from beekeepers that were present in natural areas and their bees depended on the natural plants of the region. Identifying and determining the frequency of plant families that are effective in the antioxidant activity of honey showed that their distribution and frequency depend on their coordinates.

## Materials and methods

### Introduction of the study area

**Figure 1:**
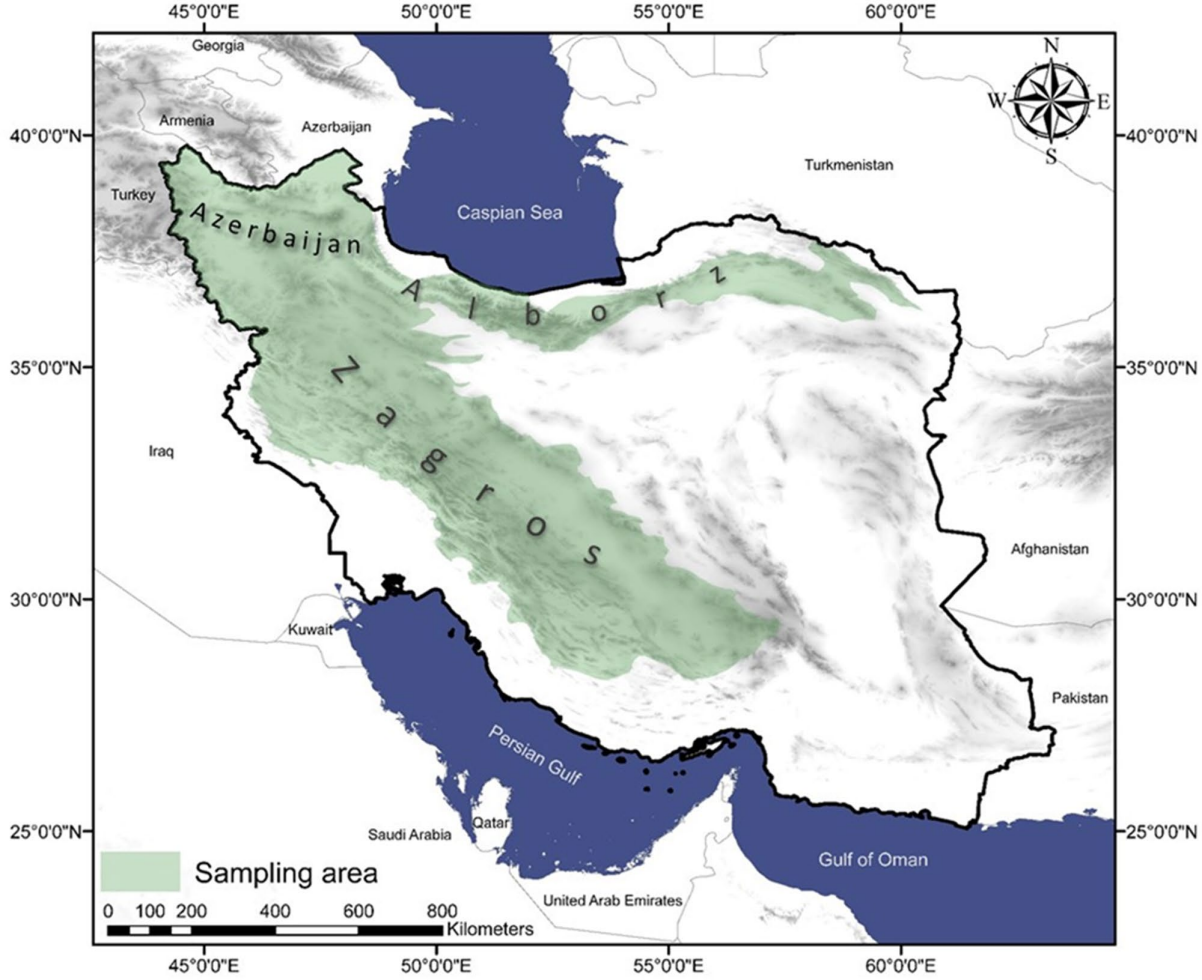
Study area.

Due to its geographical and topographical conditions, Iran is home to about 8200 plant species, of which more than 2300 species are known as medicinal and aromatic plant species^49^. Zagros, Alborz, Kope Dagh and several other internal mountain ranges are considered as the main geomorphological regions of Iran. The Zagros mountain range extends from the northwest to the southeast, and the Alborz mountain range is located at 36 degrees north of the Caspian Sea with a height of 5671 m^50^. The Zagros and Alborz mountain ranges and the highlands of Azerbaijan in Iran have formed valuable plant communities with natural plant species, some of which are endemic. Alborz and Zagros are the habitats of many 2597 native vascular plant species in Iran^51,52^. The diverse plant communities of Zagros and Alborz and the highlands of Azerbaijan have turned them into hubs of honey production in Iran^48^. Therefore, these areas were chosen to collect honey samples (Fig. 1).

### Sample collection

Samples were collected in 2020–2021 directly from beekeepers that were present in natural areas and their bees depended on the natural plants of the region. The geographic coordinates of each sample were recorded separately by a Garmin Map64s GPS device. Each collected sample contained 300 g of unrefined and non-clay honey from each colony. Each sample was coded separately and placed in a sterile container and transported to the laboratory at a temperature of < 20 °C. In this study, honey samples were collected from 94 different regions in Iran.

### Melissopalynological analyses

Louveaux method was used in this study to analyze Melissopalynology^53^. The International Commission on Bee Botany (ICBB) has proposed the melissopalynological method^53,54^. Pollens were extracted from honey samples according to the Louveaux method. First, 10 g of honey in about 20 mL of distilled water was dissolved on a hot plate at a temperature below 40 °C. Then, the honey samples were filtrate to remove large honey wax particles, the liquid was poured into 30 mL conical centrifuge tubes. After several centrifugation times (2500–3000 rpm) at room temperature (21–25 °C), pollen grains were extracted from the honey. In the first round, the samples were centrifuged for 10 min (approximately 2500 rpm) and the supernatant was carefully removed to avoid pollen loss. The remaining precipitate was mixed again with about 10 mL of distilled water, but this time the samples were centrifuged for 5 min (2500–3000 rpm). After the last centrifugation, we poured the last remaining liquid into the watch glass and placed it on the hot plate so that the water evaporated completely and the pollen grains remained at the bottom of the container. We placed the prepared pollen grains on special bases and, after coating the samples with gold metal, we placed them inside the scanning electron microscope (SEM) and then the pollen grains obtained from honey sediments were counted using a SEM. In order to determine the relative abundance, the number of 500–1000 pollen grains should be identified in each honey sample^55^. Pollen grains were identified based on their morphology using atlases and international palynology websites.

### Chemical analyses

Antioxidant activity was measured based on 2,2-Diphenyl-1-picrylhydrazyl (DPPH). All honey samples were analyzed using DPPH free radical scavenging activity by antioxidant activity analysis^56^. Honeys were diluted 1/1 (w/v) with distilled water. DPPH solution was prepared by dissolving 0.1 mM of DPPH in methanol. In its radical form, DPPH solution with purple color shows an absorbance maximum at 515 nm which disappears upon reduction by an antiradical (anti-oxidant) compounds which turn its purple to yellow color. Ascorbic acid (20 mg/mL) was used as a positive control. Ascorbic acid was used as a strong antioxidant compound, which in this concentration can completely reduce DP and cause the maximum decrease in DP absorption at 530 nm. This mode can help in reducing absorption in DPPH when comparing honey samples. To measure the antioxidant activity of the honey samples, 10 μL of diluted honey was mixed with 100 μL of DPPH and incubated for 30 min in the dark. Absorbance was recorded using a spectrophotometer at a wavelength of 520 nm. The control sample consists of 10 μL of water and 100 μL of DPPH.

Antioxidant activity was calculated using the following formula

$$DPPH\;scavenging\;activity\;(\% ) = A_{control} - A_{sample} /A_{control} \times 100.$$

### Instrumentations and reagents

Hitachi SU3500 scanning electron microscope (SEM) made in Japan.

Spectrophotometer JENUS V-1100 made by DLAB China.

Ascorbic acid and 2,2-Diphenyl-1-picrylhydrazyl (DPPH) were obtained from Sigma and Merck Chemical Company respectively.

### Statistical analysis

To determine the degree of dependence of antioxidant activity on plant families, between the abundance of each family and their antioxidant levels, regression was taken by Fitted line plot, Minitab19 with a confidence level of 95%. ArcGIS 10.8 software was also used to describe the abundance of effective plant families and the amount of antioxidant activity of honey samples.

### Statement

In the above study, the beekeepers provided us with honey samples voluntarily and with full consent, and we did not need to obtain a permit due to not entering protected areas or national parks.

## Results

**Figure 2:**
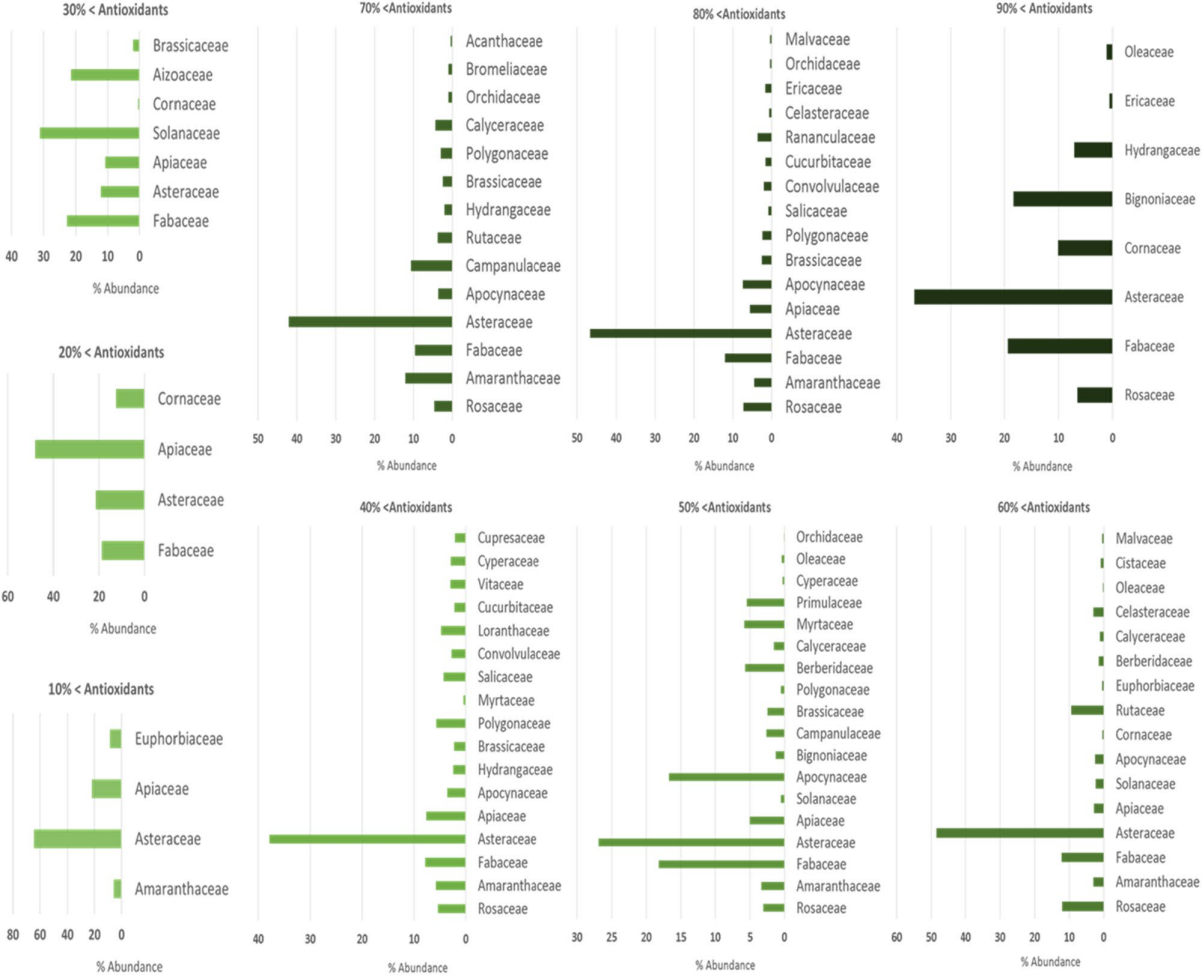
Abundance of plant families identified from pollen extracted from honey samples.

Of the 94 collected samples, it was not possible to identify pollen in 37 samples, and for this reason, it was excluded from the melissopalynology part of the study. From the studied honey samples, 42 plant families from 55 plant genera were identified and their frequency was determined (Fig. 2).

**Figure 3:**
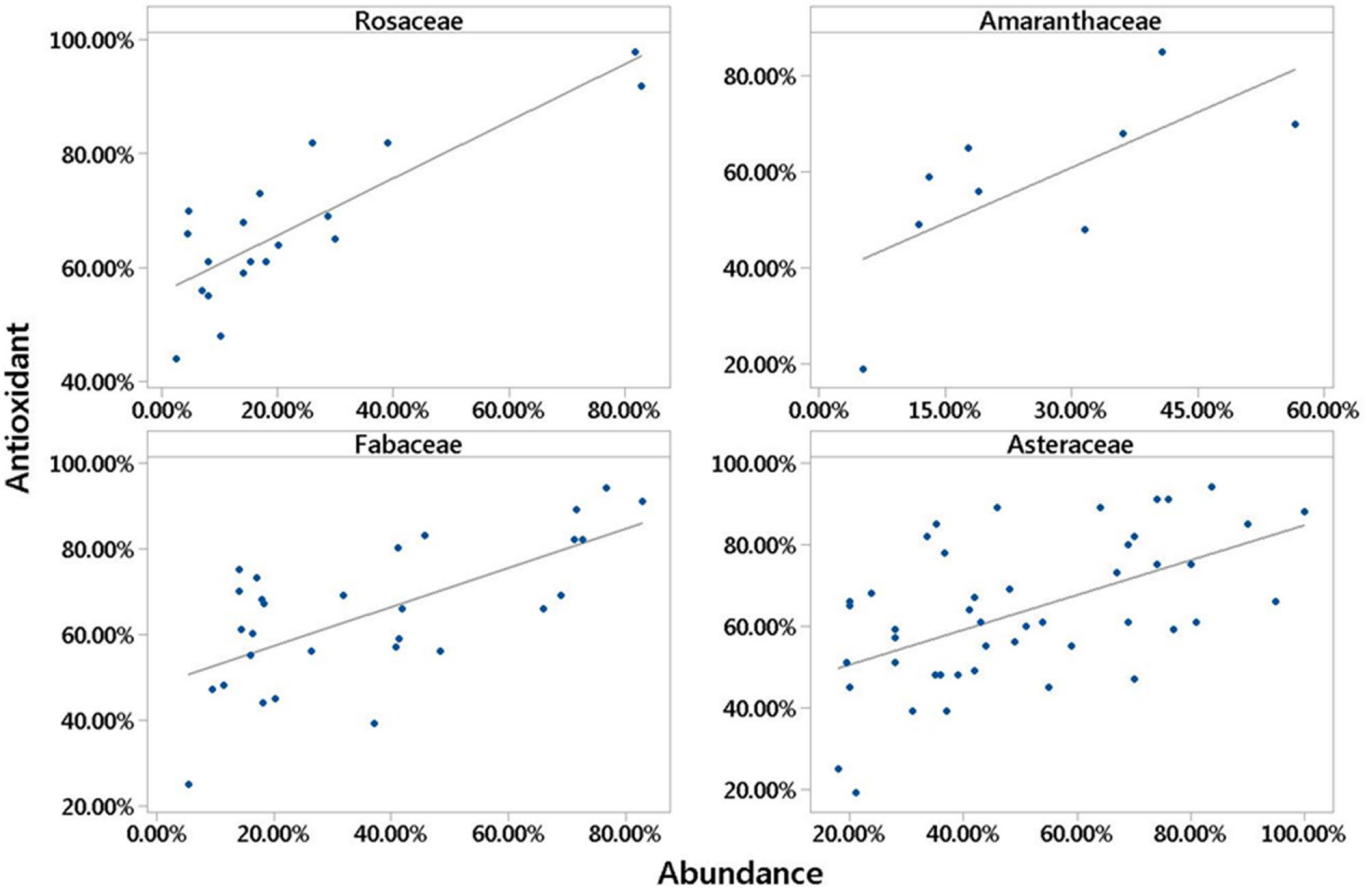
Plant families whose abundance increases antioxidant activity in honey.

Among the number of identified plant families, 4 families Rosaceae (p 0.000, R^2^ > 62%), Amaranthaceae (p 0.040, R^2^ > 47%), Fabaceae (p 0.000, R^2^ > 45%) and Asteraceae (p 0.000, R^2^ > 31%), were respectively the most effective families in the amount of antioxidant activity of honey. The result showed that the increase in abundance of plant families Rosaceae, Asteraceae, Amaranthaceae and Fabaceae in honey has increased the amount of antioxidant activity of the studied honey samples (Fig. 3).

**Figure 4:**
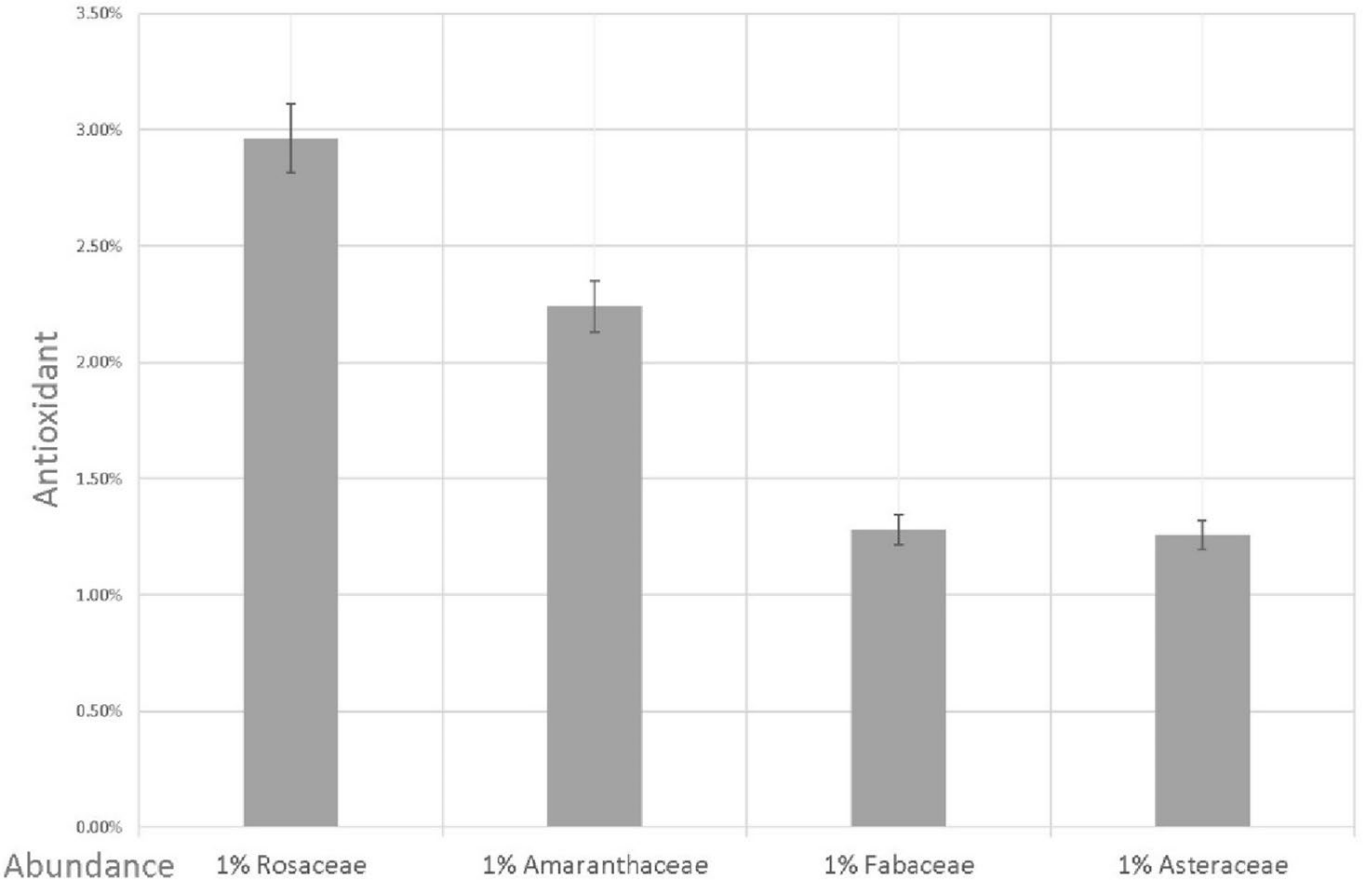
The influence of the presence of 1% of each plant family on the antioxidant activity of honey.

The four identified families effective on the amount of honey antioxidants have different influence power. As Fig. 4 shows, for 1% of the presence of Rosaceae family in honey, it causes an rise of about 3% of antioxidants, which shows more effective power compared to other samples (Fig. 4).

**Figure 5:**
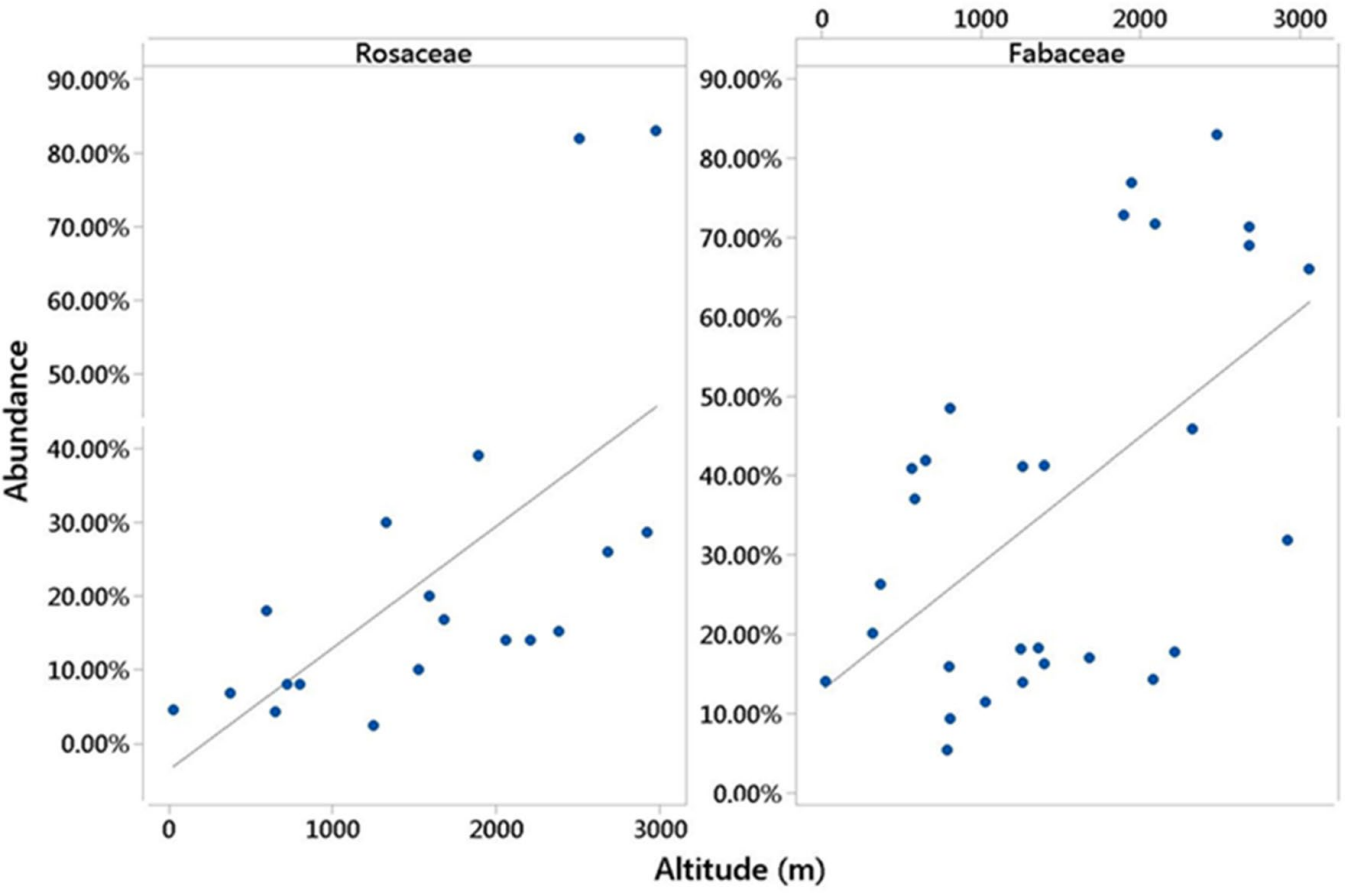
The abundance of Rosaceae and Fabaceae families in honey shows an increasing trend with rise altitude up to 3000 m. The four families influencing the amount of antioxidant activity of honey were sampled throughout the region and showed different frequencies. Figure 6 describes the abundance of four families in the collected honey samples.

The abundance of Rosaceae (p 0.003, R^2^ > 41%) and Fabaceae (p 0.001, R^2^ > 31.7%) families showed a significant increase with the rise in the altitude of the beekeepers' settlement. Also, the results showed that the abundance of the Rosaceae and Fabaceae family in the collected honey samples had a direct relationship with the increase in altitude (Fig. 5).

**Figure 6:**
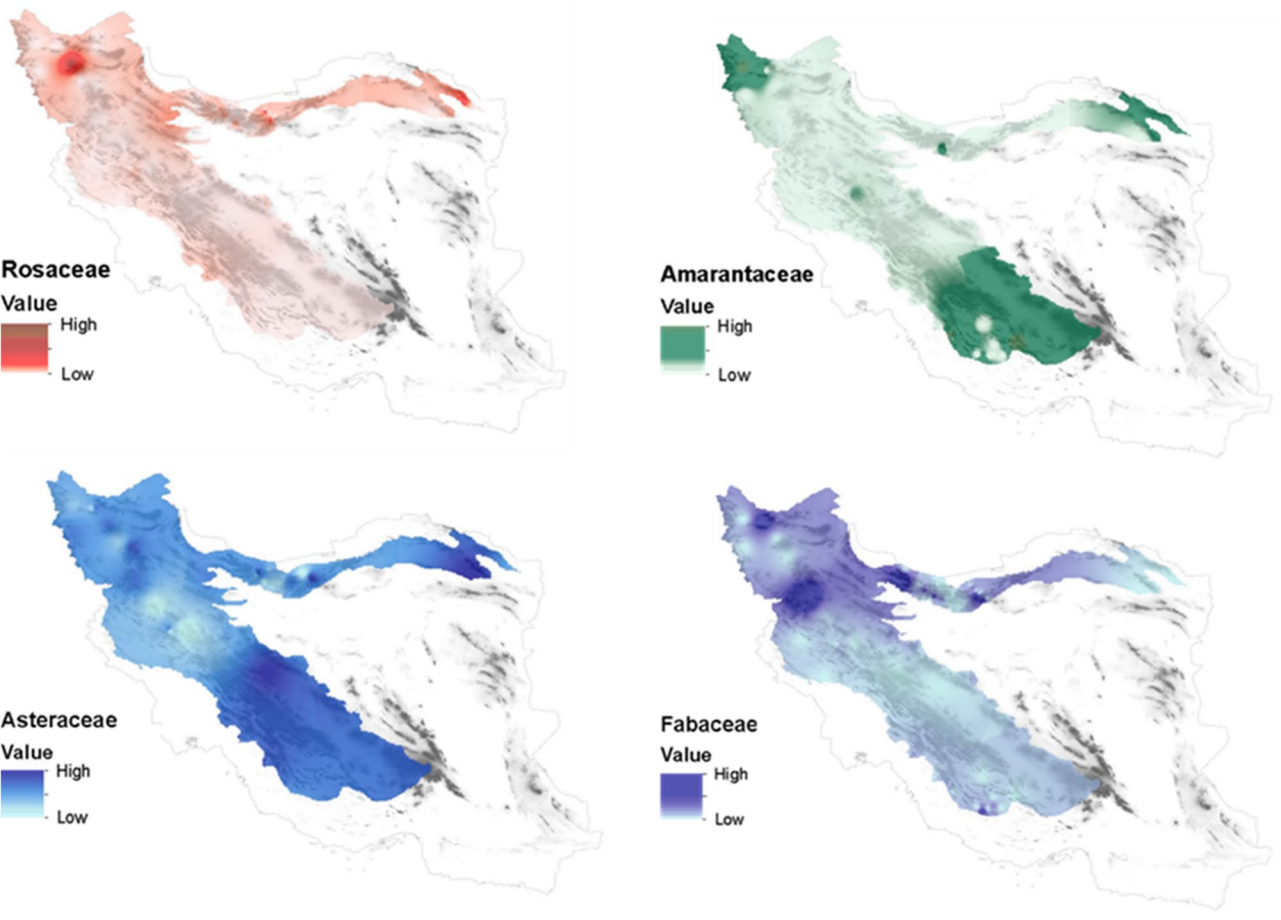
Abundance of plant families affecting antioxidant activity in honey.

**Figure 7:**
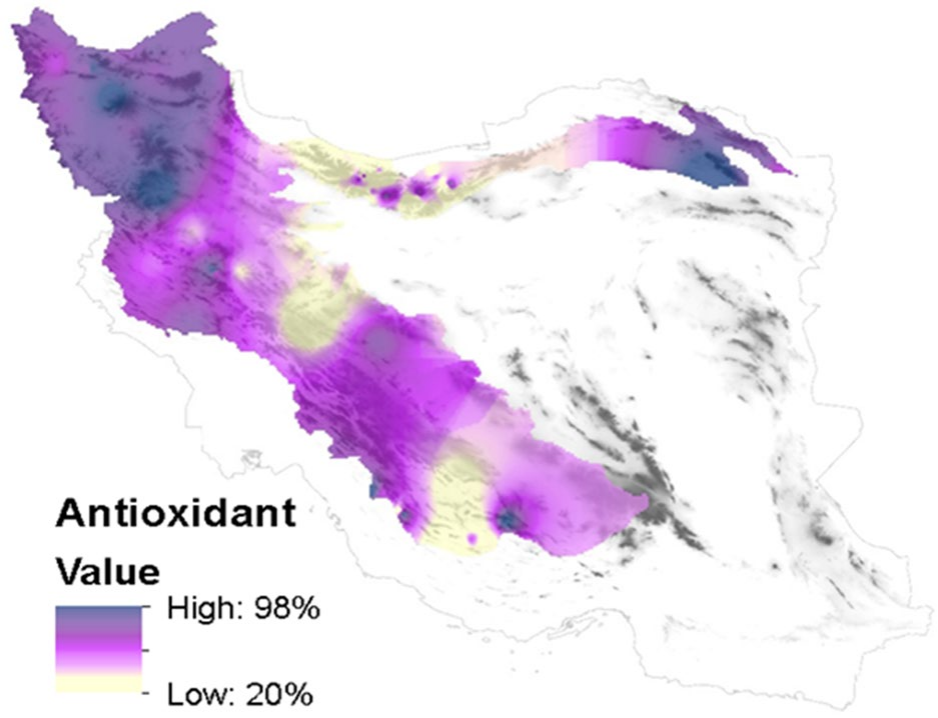
The range of antioxidant activity of honey in the sampled areas.

Antioxidants of the studied samples showed that geographical coordinates can act as an important determining factor in the amount of honey antioxidants. Figure 7 describes the amount of antioxidant activity of honey geographically in the sampled area.

## Discussion

This study was conducted in order to identify effective plant families in determining the antioxidant activity of honey. Also, the results of this study compared the influence of effective families on the amount of antioxidant activity of honey. So that for 1% abundance of Rosaceae family, the antioxidant of honey can increase by about 3%. The results of this study showed that the height and abundance of Rosaceae and Fabaceae families have a direct relationship, so that the abundance of these two families increased with the rise in attitude. The geographical part of the results of this study showed that the abundance of four plant families in honey is not uniform and in some areas, each of them shows a high relative abundance. Also, the amount of antioxidant activity of honey varied from 98 to 19% in the sampled areas.

The results of this study showed that the Rosaceae family has the greatest effect in increasing the antioxidant activity of honey because the plant species of the Rosaceae family are considered an important natural source of antioxidants^57^. Also, increasing the abundance of plant species of the Fabaceae family in honey compared to other families has a greater impact on increasing the antioxidant activity of honey^18^. The results of studies have shown that almost all components of the Rosaceae family are considered as an important source of natural antioxidants^58,59^.

The results showed that the Amaranthaceae family is an effective family on the amount of antioxidants in honey. With a 1% rise in the abundance of Amaranthaceae family in honey, its antioxidant activity increased by about 2.3%. The Amaranthaceae family, having a significant amount of natural antioxidants, has turned this family into an important plant family for medicinal purposes^29,60,61^.

The Fabaceae family was identified as an important family in determining the antioxidant content of honey in this study, which combined melissopalynology studies, confirms our results^62^. An increase in the abundance of the Fabaceae family by 1% rise the antioxidant activity of honey by about 1.3%, because molecular studies have shown that the species of the Fabaceae family have an effective and significant amount of antioxidants^63–65^. The amount of antioxidants of some species of the Fabaceae family is so impressive that some studies have suggested for the prevention and treatment of various cancers^66,67^. Also, the Fabaceae family plays an effective role in increasing the amount of antioxidants in honey.

The Asteraceae family is the fourth identified family in this study, the increase in its abundance is directly related to the rise in the antioxidant activity of honey. The significant amount of antioxidant activity in the plant species of the Asteraceae family has made this plant family an important medicinal plant family in the studies of prevention and treatment of various diseases^68,69^. The results of this study showed that a 1% rise in its frequency causes an increase of about 1.3% in the antioxidant activity of honey. Some studies confirm the effect of the presence of the Asteraceae family on the amount of antioxidants in honey^70,71^.

The ecological results of this study showed that the abundance of Rosaceae and Fabaceae families, which play an effective role in the antioxidant activity of honey, rise with increasing altitude. Therefore, it seems that the increase in antioxidant activity due to the rise in altitude up to 3000 m above the surface of open water^48^ is largely influenced by the abundance of these two families. For example, the Rosaceae family grows in high areas^72^. Therefore, there is a direct relationship between the plant-geographical origin of honey production and its biological properties^73,74^. Also, the contents of medicinal and aromatic plants show significant changes depending on the region and climatic conditions^27^.

## Conclusion

The antioxidant activity of honey has a direct and significant relationship with the plant and geographical origin of honey, and the presence of some plant families significantly increases the antioxidant activity in honey. The four plant families Rosaceae, Amaranthaceae, Fabaceae, and Asteraceae, respectively, in this study had the greatest influence on the amount of antioxidant activity of honey compared to other plant families. The abundance of two plant families, Rosaceae and Fabaceae, which play an effective role in the antioxidant activity of honey, also increases compared to altitude. Many species of the four plant families effective in the amount of antioxidants in honey are native and endemic species of the Iranian plateau. Therefore, these areas have the potential to produce honeys with high antioxidant activity, which has made the protection of plant communities in these areas important.

## Data Availability

Datasets analyzed during the current study are available on Figshare as https://figshare.com/s/cc9d170a8530fb798e26.
